# Assessing the Relationship between Socioeconomic Conditions and Urban Environmental Quality in Accra, Ghana

**DOI:** 10.3390/ijerph7010125

**Published:** 2010-01-13

**Authors:** Julius Fobil, Juergen May, Alexander Kraemer

**Affiliations:** 1 Infectious Disease Epidemiology Unit, Bernhard Nocht Institute for Tropical Medicine, Bernhard-Nocht-Str. 74, D-20359 Hamburg, Germany; E-Mail: may@bnitm.de; 2 Department of Public Health Medicine, School of Public Health, University of Bielefeld, P.O. Box 100131, D-33501 Bielefeld, Germany; E-Mail: alexander.kraemer@uni-bielefeld.de; 3 Department of Biological, Environmental, Occupational Health Sciences, School of Public Health, University of Ghana, P.O. Box LG13, Legon, Ghana

**Keywords:** Ghana census data, area-based SES, Accra, socioeconomic inequalities

## Abstract

The influence of socioeconomic status (SES) on health inequalities is widely known, but there is still poor understanding of the precise relationship between area-based socioeconomic conditions and neighborhood environmental quality. This study aimed to investigate the socioeconomic conditions which predict urban neighbourhood environmental quality. The results showed wide variation in levels of association between the socioeconomic variables and environmental conditions, with strong evidence of a real difference in environmental quality across the five socioeconomic classes with respect to total waste generation (*p* < 0.001), waste collection rate (*p* < 0.001), sewer disposal rate (*p* < 0.001), non-sewer disposal (*p* < 0.003), the proportion of households using public toilets (*p* = 0.005). Socioeconomic conditions are therefore important drivers of change in environmental quality and urban environmental interventions aimed at infectious disease prevention and control if they should be effective could benefit from simultaneous implementation with other social interventions.

## 1. Introduction

**Figure 1.**
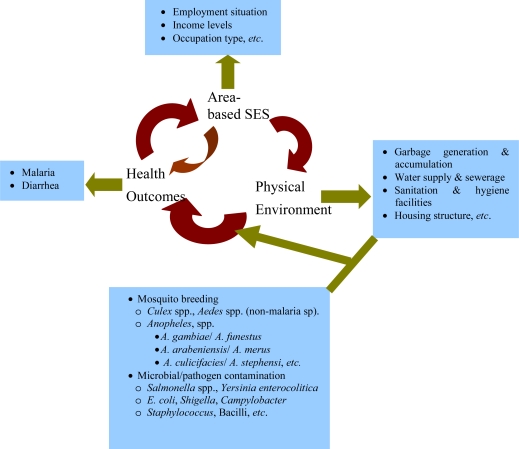
Interaction among Area-base SES, Environmental Quality and Health.

The influence of socioeconomic factors on health outcomes has long been recognized and past research effort has focused on the relationship between socioeconomic status (SES) and health inequalities among different subpopulation groups [1]. SES is frequently implicated as a contributor to the disparate health observed among racial/ethnic minorities, women and elderly populations [2–5]. There is scientific consensus that several factors (both SES and the physical environment, see Figure 1) interact to influence health status and health disparities among populations [1–3,6–8]. In U.S., SES is among the factors most frequently implicated as a contributor to the disparities in health observed among populations [1,4]. Other factors include lifestyle, the cultural and social environment, living and working conditions as well as social and community networks [9,10]. Adler and coworkers modeled three pathways through which SES impacts health, which include its association with healthcare, environmental exposure, and health behavior and lifestyle [11]. In Figure 1, a simplified theoretical model of SES, environment and health interaction is presented to show the interlinkages among the constructs.

Generally, health inequalities exist among rural and urban dwellers, different incomes groups, different gender and age-groups in developing countries. The dependence on cash-for-service policies in many African and other low- and middle-income countries has increased inequalities in access to affordable health care which tend to produce disparate health outcomes among different social groups. Wide inequalities in income levels also mean uneven access to environmental services which drive environmental health inequalities across these social groups. In literature, many studies exist which highlight health problems of the urban population in the cities of Africa, Asia and Latin America [5,11–13]. Intra-urban differentials in social, environmental and health conditions between groups in cities are now broadly understood [2,3] and depending on the region, between 35 and 55 percent of the population in developing countries including those in Africa have incomes or consumption levels below the standard poverty line [4,9,14,15]. While urban poverty is rapidly exacerbating, a marginally small but numerically consequential proportion of urban residents have lifestyles and living conditions which mirror those of the very affluent countries [5,16–18]. Several review articles have reported widening intra-urban differentials in environmental quality conditions in the poor countries [2,4–7,9,10,16–23]. In Ghana, such reviews and assessments reported pervasive intra-urban environmental quality differentials in the fast growing urban centers including Accra, Kumasi, Tamale, Cape Coast and Takoradi, where deprived areas exist alongside privileged areas, distinguished only by the overall area-based socioeconomic conditions [12,24–27]. In Accra, up to 46 percent of people live in the most deprived zones [24,25,27]. These areas accommodate people with the lowest educational standards, the lowest incomes and the poorest facilities in terms of water, sanitation and housing [24,25,27].

Although analysis of data on socioeconomic status has nearly always been included in epidemiologic research, its specific use is often dependent on data availability [1,4,9,23]. While it is often concluded that differences in SES are the cause of differences in health outcomes among population groups, there is often little, if any, discussion of the specific manner in which SES exerts its influence within the context of the study outcomes [28–30]. This then leaves a gap regarding the chain of events leading from the multiple pressures from neighborhood socioeconomic conditions driving changes in neighborhood environmental conditions, which then directly influence health outcomes, see Figure 1. These neighborhood urban environmental conditions are understood to constitute breeding media (Figure 1) for many infectious disease vectors including *Anopheles gambiae* – an insect vector for *Plasmodium falciparum* which causes malaria [31,32]. Household refuse (solid wastes) if not properly discarded may create routes for transmission of microbial agents [33–35]. Many insect species are known to be mechanical vectors of infectious diseases, especially diseases associated with filth [36,37]. For instance, the housefly, has sensory organs able to sense decomposing organic materials and the odor emanating from refuse dumps [33,36–38]. Additionally, uncollected or improperly managed solid wastes become receptacles of large quantities of human excreta e.g., dump diapers, faecal matter, *etc.*, may be washed into refuse dumps by torrential rains [36,39]. Excreta may also be washed during flooding into nearby wells, streams, both underground and surface water bodies leading to microbial contamination of these water bodies [37]. As a consequence, deteriorating urban environmental quality in most cases tends to increase infectious disease transmission rate [33,35–37].

In epidemiological studies, experimental designs almost always aim at finding out whether observed differences in health outcomes among study subjects or groups are indeed real differences or may merely be due to chance [30,40–42]. However in more complex study settings such as ecological designs, the existence of confounders and effect modifiers (intervening physical environmental media) do not lend easy interpretation of findings from studies which aim to look at the influence of SES variables on health outcomes. In other words, evaluation of the influence of SES on health disparities is difficult to achieve realistically without first understanding the influence of these variables on the physical environmental media/conditions.

Secondly, the precise role of SES variables in determining the observed health outcomes in populations is not clearly defined *i.e.,* whether these factors themselves alone directly influence health outcomes (e.g., issues of economic barriers to healthcare) or they do so through an intermediate (e.g., intervening physical environmental media) [4,7,43]. For instance, how are the different area-based socioeconomic factors associated with urban neighborhood environmental quality conditions? Additionally, it is not exactly clear how much influence each area-based SES exerts on the observed neighborhood urban environmental quality conditions.

Consequently, given the amount of spurious effects SES variables cast upon environment and health analysis, it becomes a worthwhile undertaking to investigate the precise nature of the effects which the different SES variables exert on environmental variables in urban settlements, *i.e.,* what is the precise nature of the association between the different area-based SES variables and the urban environmental conditions?

Although it must be acknowledged that no standard measures of the concept of SES exist and there is only very little agreement in the literature on its definition and the exact measurement of the concept, construction of proxies of the SES variables is possible and already widely applied in SES and health inequality research [1,28,44]. For instance, in the absence of individual level data on social backgrounds, area-based measures of socioeconomic status are often constructed based on social and economic aspects of the area in which the person resides. In Australia where this technique has already been widely applied, the units of measurement have been based on postcodes, Statistical Local Areas, Local Government Areas and Census Collection Districts. For the purposes of construction of area based SES measures, we adopted Census Collection Districts (Census Clusters) of the Ghana Statistical System (GSS) as the units of analysis.

The aim of this study was to achieve the following:

- to determine the kind of association between area-based SES conditions and the quality of neighborhood urban environmental conditions,
- to determine the amount of variability in urban neighborhood environmental conditions that can be explained by area-based socioeconomic factors,
- to assess the levels of environmental health inequalities across urban socioeconomic landscape, and
- to find out if there are differences in the quality of the neighborhood urban environmental conditions across the different wealth quintiles.

## 2. Methodology

### 2.1. Study Area

This research was conducted in Accra, the capital city of Ghana; a small country located on the Atlantic Coast of West Africa. The country occupies a total land area of 238,537 square kilometers and has a total population of 18.9 million [45–52]. Greater Accra Region, where Accra is located, is the smallest (in terms of land surface area) of the ten political regions in Ghana. It is however the largest (in terms of population size) of Ghana’s ten leading urban centers, with an approximate population of 1.7 million in 1990 and 2.7 million in 2000 [12].

Accra harbors over 30% of the urban population and nearly 15% of the country’s total population. The generation and annual rate of increase of solid waste is high in Ghana and in the capital city of Accra, *per capita* production of refuse is estimated at 0.40 kg/person/day [53–55]. Nearly 60% by weight of this huge chunk of waste generated is organic material; representing 0.3 million metric tons of waste annually and over 50 percent of the solid waste generated is left uncollected [54] which allows for high waste deposition rate. The general topography of the city is flat low-lying terrain, underlain with clayish and impervious soils and characterized by inadequate and undersized drains. The flat terrain is drained by the Odaw River and the Korle River and dotted at several points by lagoons, swamps, large drains, ponds and other water bodies which are strewn with and/or polluted by both solid and liquid wastes [13,55]. As a consequence of rapid urbanization, there are imbalances in the provision of basic sanitation services which have left the city to form clusters at different levels of environmental quality conditions [56]. Key problems facing the city are rapid waste deposition, citywide filth and systemic deterioration in urban environmental conditions as well as a general decline in aesthetic beauty [12,13]. The city consists of six sub-metro districts which for census enumeration purposes has subdivided into 70 census clusters [12].

### 2.2. Study Design and Data Collection

For the purposes of this study, not only geographically contiguous Enumeration Areas (EAs), but also EAs with similar population characteristics were merged to produce census clusters (the units of the analyses). This was based upon the census cluster definition by the statistical system of Ghana as a group of geographically contiguous census enumeration areas of fairly homogeneous populations according to defined area characteristics such as accessibility of population to enumerators, socioeconomic conditions, cultural factors, *etc.*, [49–51]. The boundaries of these clusters were digitized to produce polygons of the census clusters and which were pieced together to produce a complete digital map of urban Accra [50]. The Accra metropolis consists of 1,700 EAs [45,50] which after the process produced 70 census clusters. Five (5) distinct wealth quintiles; viz poorest class, lower middle class, middle class, upper middle class and high class, were constructed from the unidimensional measure [1,11,57]. A comparison of environmental quality conditions in the different wealth quintiles was then undertaken. The neighborhood environmental measures included in this analysis were total solid generation, per capita waste generation, waste collection rate, waste uncollected (deposition) rate, sewer disposal rate, non-sewer disposal rate, proportion of households with pit-latrines, proportion of households with bucket/pan latrines, proportion of households with toilet/bath facility outside and proportion of households using public toilets. Both the socioeconomic and environmental variables were obtained from the census 2000 database at the Census Secretariat of the Ghana Statistical System (GSS) by written permission of the Government Statistician.

### 2.3. Area-based Socioeconomic Variables

The 2000 census database held several cluster level measures of socioeconomic status including educational attainment, literacy rate, school enrolment, religion, ethnicity, marital status, employment status, type of employment, place of employment, economic activity status (e.g., whether employable or not, *etc.*). There were 53 of these socioeconomic variables in total (Appendix 1) which were obtained already grouped by the GSS under six main categories as:

- economic activity status
- educational attainment
- occupation
- place of work
- marital status, and
- ethnicity.

The grouping was done based on the criteria set out in the Ghana Living Standards Survey (GLSS) framework [24,25,27,48–50]. In this study, marital status and ethnicity were excluded because they were perceived to be politically and culturally sensitive. We explored the remaining variables using Principal Component Analysis (PCA) to determine their relationships with each other, the direction of the eigen vectors and to be able to develop a uni-dimensional measure of SES, e.g., socioeconomic zones (quintiles) for the study area. The variables used in constructing the area-based socioeconomic measures were computed as a proportion of individuals with a given socioeconomic characteristic among the total number of individuals in a cluster. These area-based measures were used as proxies for cluster level socioeconomic conditions in lieu of the traditional or conventional measures of SES which are based upon household incomes, asset-based indices, consumption or expenditure indices, *etc.*, because they can be measured more reliably compared to their traditional counterparts. For instance, while most people will feel reluctant to talk about incomes and earnings, often forget household expenditures and may not be reporting correct income levels, it is fairly easy to accurately count the number of unemployed *vs*. employed or economically active *vs*. economically inactive people in a survey. For this reason, the measures of economic status adopted in this study seem more reliable compared to the conventional ones.

### 2.4. Physical Urban Environmental & Neighborhood Quality Conditions

Data on urban environmental quality conditions were in similar manner obtained from the Ghana Statistical Service (GSS) [50]. The environmental (response or outcome) variables of interest in this analysis were computed into proportions of the total cluster level conditions (Table 1) according to existing well defined categories as below.

Cluster level urban water supply, hygiene and environmental sanitation quality was estimated broadly under the following measures:

○ per capita waste generation
○ total waste generation
○ proportion of solid wastes collected
○ proportion of solid wastes uncollected (waste deposition)
○ proportion of liquid wastes by sewer disposal
○ proportion of liquid wastes by non-sewer disposal
○ proportion of households with pit-latrines
○ proportion of households with toilet/bath facility in different house
○ proportion of households with pan-latrines,
○ proportion of households using public toilets.

### 2.5. Analytical Approach

In this analysis, PCA was used to develop wealth quintiles for urban Accra. From the exploratory analyses, a factor score with a low absolute value represented low SES and that with high absolute value indicated high SES (Appendix 1). A thorough assessment of whether there were differences in neighborhood urban environmental quality conditions across the socioeconomic classes (*i.e.,* the wealth quintiles developed) was conducted. Finally, the area-based socioeconomic variables were employed in multiple linear regression models as explanatory variables to explore the association between cluster level socioeconomic conditions and the cluster level neighborhood urban environmental quality conditions.

Appendix 1 shows all the area-based socioeconomic variables that were obtained from the 2000 census database, their mean proportions, standard deviations and eigenvectors (factor scores). An initial exploration using PCA was conducted on all the variables to determine the direction of their influence on SES or human wellbeing and to reduce the large number of variables to a manageable uni-dimensional variable [57]. Those variables which had strong loading (*i.e.,* those with factor scores equal/greater than 0.3 or equal/less than −0.3) were retained while those with poor loading were excluded in the final PCA model that was used to develop the uni-dimensional measure. In the initial PCA model, 39 variables were included. Out of the 39 variables, 16 variables exhibited strong loading (Appendix 2). The 16 SES variables were employed in the final PCA model to construct a unidimensional measure from which socioeconomic quintiles were developed for urban Accra (Table 1). The final output from the PCA model showed 16 corresponding components with component 1 (comp1) explaining 33.9 percent of the variation in socioeconomic conditions (Appendix 3). Overall, five components (*i.e.,* comp1, comp2, comp3, comp4 and comp5) were significant and accounted for up to 82.4 percent of the total variation in the socioeconomic conditions. However, in constructing the socioeconomic classes, we relied solely upon comp1 which was responsible for the largest variation in the overall socioeconomic conditions, *i.e.,* accounted for more than 30 percent of the total variation (Appendix 3) [57].

## 3. Results

Table 1 shows the variation in neighborhood urban environmental quality conditions across socioeconomic classes in a typical urban setting in a low-income economy. In general, while there was very strong evidence of differences in the levels of environmental quality with respect to total waste generation (*p* < 0.001), waste collection rate (*p* < 0.001), sewer disposal rate (*p* < 0.001), non-sewer disposal (*p* = 0.003) and the proportion of households using public toilets (*p* = 0.005), only moderate evidence of a difference in the environmental quality was observed for per capita waste generation rate (*p* < 0.015) and the proportion of households with toilet/bath facilities outside own household (*p* = 0.02) across the socioeconomic classes.

With respect to inter-quintile variability, whereas there was no evidence of differences between the poorest class and the lower middle class for total waste generated (*p* = 0.064), per capita waste generated (*p* = 0.103) and the proportion of waste collected (*p* = 0.403), there was very strong evidence of a difference across the higher wealth quintiles.

For instance, a very strong evidence of differences in neighborhood urban environmental quality conditions was observed across the wealth quintiles; *i.e.,* the lower middle class and middle (*p* = 0.002), middle class and the upper middle class (*p* < 0.001), the upper middle class and the high class (*p* = 0.004) for the amount of waste generated at cluster level. For per capita waste generation, the weight of the evidence of differences was equally very strong *i.e.,* the lower middle class and middle (*p* = 0.001), middle class and the upper middle class (*p* = 0.014), the upper middle class and the high class (*p* = 0.010). Similar trend was observed for waste collection rate at cluster levels. There was even much stronger evidence of a difference across the wealth quintiles for uncollected waste (deposition rate), sewer disposal rate, non-sewer disposal rate and the proportion of households relying upon facilities outside households and public toilets (Table 1). Although, there were differences in the levels of inter-quintile variability of the different urban environmental quality conditions, the weight of the evidence; except for the proportion of households with pit and bucket/pan latrines, was generally strong (Table 1), suggesting a strong link between area-based SES and urban neighborhood environmental quality conditions.

In the next stage of the analysis, a key interest was also in how multiple factors influenced the overall neighborhood environmental quality. This meant that, it was desired to assess the relationship between area-based SES and neighborhood urban environmental quality conditions. For example, per capita solid waste generation was regarded as an important urban environmental quality measure as it was the basis for calculating the total amount of solid waste a given population generated per unit time and often the basis of waste management planning programs (e.g., size of sanitary landfills to construct, type of tipping-trucks to import, financial capital required for solid waste transport, *etc.*). Authors used bivariate and multiple regression techniques to assess such relationships.

There was a positive (*i.e.,* a unit increase in population economic inactivity resulted in an increase in per capita solid waste generation rate) association between the proportion of economically inactive cluster population (economic inactivity) and per capita solid waste generation (regression coefficient = 0.276) and the amount of variation explained by economic inactivity was 3.5 percent (R^2^ = 0.0346). Economic inactivity measures the number of economically inactive residents within a given self-sustaining resident urban population who were technically dependent on economically active residents for social support and this measure was computed separately for males and females.

Despite this marginal increase, there was no evidence of association between economic inactivity and per capita solid waste generation (*p* = 0.13; 95%CI: −0.079–0.631). Additionally, a sex-stratified analysis of the economic inactivity or any of the remaining SES [*i.e.,* for male (*p* = 0.50), and for female (*p* = 0.40)] found no evidence of association with the neighborhood urban environmental conditions. The amount of variation in neighborhood urban environmental quality conditions explained by variation in each of the two SES measures separately was less than 3 percent.

However, there was an inverse association (*i.e.,* unit increase in economic activity led to a decrease in per capita solid waste generation) between economic activity and per capita solid waste generation (regression coefficient = −0.276) and the amount of variation explained by economic activity was 3.5 percent (R^2^ = 0.0346), essentially the same as the amount of variation explained by economic inactivity.

Further analysis showed a moderate positive (a unit increase in urban employment rate led to a slight increase per capita solid waste generation rate) association between urban employment rate and per capita solid waste generation rate (regression coefficient = 0.566) and the amount of variation in per capita solid waste generation rate that was explained by urban employment was 4.2 percent (R^2^ = 0.042). There was no evidence of association between urban unemployment and per capita solid waste generation rate (*p* = 0.09; 95%CI: −0.093–1.224).

Additionally, a positive (regression coefficient = 0.884) association was observed between urban employment and urban solid waste collection rate. The amount of variation explained by urban employment was 6.2 percent (R^2^ = 0.062). There was a moderate evidence of association between urban employment and urban solid waste collection rate (*p* = 0.039; 95%CI: 0.046–1.721).

**Figure 2.**
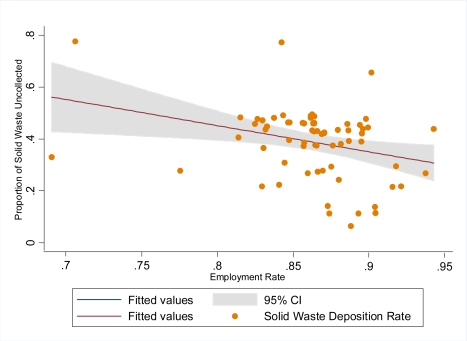
Variation of employment rate with proportion of solid waste deposition rate.

Figure 2 depicts the relationship between urban employment rate and urban solid waste deposition rate. An inverse (regression coefficient = −1.007) was demonstrated and the amount of variation in solid waste deposition rate that was explained by urban employment was 9.5 percent (R^2^ = 0.095). As shown, a unit increase in the proportion of urban employment resulted in a significant decrease in urban solid waste deposition rate. A very strong evidence of association was observed between urban solid waste deposition rate and the proportion of urban employment (*p* = 0.01; 95%CI: −1.764–0.250).

The relationship between urban employment and the proportion of households connected to the central sewer system (sewer disposal rate) showed a positive (regression coefficient = 0.841) association. The amount of variation in the proportion of households connected to the central sewer system explained by the proportion of urban employment was 6.4 percent (R^2^ = 0.064). This meant that a unit increase in the proportion of employed cluster population resulted in a corresponding increase in the proportion of cluster households connected to the central sewer system in the Accra metropolis. Moderate evidence of association was observed between the proportion of households connected to central sewer system and the urban employment (*p* = 0.036; 95%CI: 0.058–1.624).

**Figure 3.**
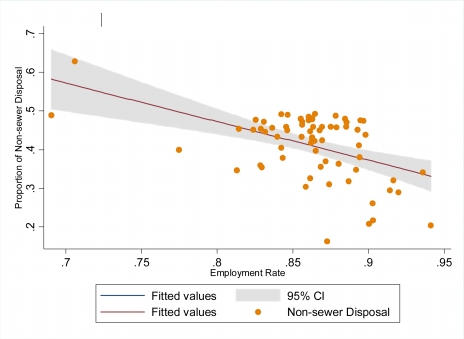
Variation of employment rate with non-sewer disposal rate.

However, an inverse (regression coefficient = −1.084) relationship was observed between urban employment and the proportion of households engaged in non-sewer (improper) liquid waste disposal (Figure 3). The amount of variation in non-sewer liquid waste disposal explained by the urban employment was 18 percent (R^2^ = 0.181). A very strong evidence of association was observed between non-sewer liquid waste disposal and urban employment (*p* < 0.001; 95%CI: −1.646–−0.521).

In contrast to the strong association between the proportion of urban households connected to the central sewer system and urban employment, no such evidence of association was observed between urban employment and such cluster hygiene conditions as the proportion of households with water closets (WC), proportion of households with pit-latrines, proportion of households with Kumasi Ventilated Improved Pits (KVIPs) *i.e.,* a locally constructed improvised community toilet, proportion of households with pan-latrines, proportion of households using public toilets, *etc*., at bivariate level. This was in contrast to what was observed at community level when the area-based socioeconomic factors were aggregated and categorized into wealth quintiles. Although the area-based socioeconomic factors exhibited no evidence of association with the neighborhood urban environmental quality conditions at the household level, strong evidence of association was observed between the area-based socioeconomic factors and urban environmental conditions across wealth quintiles at the community level.

In further multilevel analysis authors examined residents’ characteristics in relation to ability of these features to drive changes in the quality of the neighborhood urban environmental conditions. Multiple regression analysis showed no evidence of association between total waste generated and the area-based socioeconomic variables, except residents’ occupation.

In other words, educational attainment and residents’ place of work did not appear to be important factors in driving the underlying difference in the amount of wastes generated in the residential communities. Nevertheless, a few elements from residents’ occupation category showed very strong evidence of association with the amount of wastes generated in the communities *i.e.,* administrative and managerial occupations (*p* = 0.004), clerical and related occupations (*p* < 0.001), service occupations (*p* = 0.014) agriculture/husbandry/forestry/fishing/hunting occupation (*p* = 0.008), production/transport and equipment operators and laborers (*p* = 0.028), and professional technical and related workers (*p* = 0.023). In addition, the area-based SES did not show evidence of association with the amount of waste generated per person per day (per capita waste generation rate). While educational attainment and residents’ place of work showed no evidence of association, some variables which together represent residents’ occupation category showed substantial evidence of association with waste collection rate e.g., administrative and managerial occupations (*p* = 0.004), clerical and related occupations (*p* < 0.001), agriculture/husbandry/forestry/fishing/hunting occupation (p = 0.021), production/transport and equipment operators and laborers (*p* = 0.010), and professional technical and related workers (*p* = 0.044). Although education level did not show evidence of association with total waste generated, per capita generation rate and waste collection rate, residents’ educational attainment showed a very strong evidence of association between waste deposition rate (proportion of wastes left uncollected) [*i.e.,* no education (*p* = 0.005), pre-school education (*p* = 0.001), middle/JSS education (*p* < 0.001), secondary/SSS education (*p* < 0.001), vocational/technical/commercial education (*p* = 0.014) and residents with tertiary education (*p* < 0.001)]. Similarly, whereas both educational attainment and residents’ place of work showed strong evidence of association with wastes deposition rate (proportion of wastes left uncollected), residents’ occupation did not. Additionally, all but one of the 16 elements representing the residents’ occupation category showed strong evidence of association with waste deposition in the communities.

On the contrary, while educational attainment and residents’ occupation only showed moderate evidence of association with the proportion of households engaged in sewer disposal, all the elements representing residents’ place of work showed very strong evidence of association with sewer disposal rate. Both residents’ place of work and residents’ education attainment showed a very strong evidence of association with households engaged in non-sewer disposal. While the proportion of households using pit-latrine services did not show evidence of association with the area-base socioeconomic variables, two of the area-based SES; namely, residents’ education attainment and residents’ occupation showed very strong evidence of association with the proportion of households using bucket/pan latrine services. Finally, whereas only a moderate evidence of association was observed between the proportion of households using sanitation facilities in a different house and residents’ educational attainment as well as residents’ occupation, residents’ place of work showed a very strong evidence of association with the proportion of households using facilities in a different house.

## 4. Discussion

In this analysis, the association between area-based socioeconomic conditions and neighborhood urban environmental quality conditions was assessed. Often, studies which sought to evaluate the influence of socioeconomic status on health inequalities have neglected such important intermediate variables as the physical environmental conditions (environmental media), which have direct influences on health outcomes. Poor environmental quality provides condition for insect vector breeding and ultimately infectious disease transmission (e.g., mosquito, an important agent for malaria transmission, common housefly as a mechanical vector for many microbial diseases, including diarrhea, enterohaemorrhagic fever, *etc.*).

Environmental burden (e.g., local sanitation) is understood to be heavier in poor communities and declines as communities get wealthier [58]. In urban areas where consumption of goods and services per person is usually very high, residual deposition (e.g., waste production) is also very high. In rural communities, consumption of goods and services and waste production are much lower per unit compared to urban areas. However, the high consumption and high residual deposition (waste production) are not backed by equitable distribution of wealth in the urban areas thus leaving some of the urban communities financially weak to be able to manage the waste produced. In this study, the observed varied levels of influence of the area-based SES on spatial changes in the quality of the neighborhood urban environmental conditions were suggestive that the area-based SES did not exert the same degree of influence on the quality of the neighborhood urban environmental conditions. While some of the area-based socioeconomic variables were important in influencing changes in the quality of some neighborhood urban environmental conditions, they did not show any perceived influence on some other components of the neighborhood urban environmental quality conditions. For example, whereas education level did not show evidence of association with total waste generated, per capita generation rate and waste collection rate, waste deposition rate (proportion of wastes collected) was observed to be strongly associated with residents’ educational attainment.

However urban employment, urban unemployment, educational attainment, residents’ place of work and residents’ occupation have demonstrated high reliability as measures of area-based SES. The nature of the associations observed between neighborhood urban environmental conditions on the one hand and urban employment and urban unemployment on the other hand was consistent with what is already known [12,55].

Although a unit increase in urban unemployment resulted in a marginal decrease in per capita solid waste generation (regression coefficient = −0.566), there was no evidence of association between urban unemployment and per capita solid waste generation (*p* = 0.09; 95%CI: −1.224–0.093). Nonetheless, a positive relationship was observed between urban employment and per capita solid waste generation rate. In this case, once in both instances no evidence of association was observed between the two area-based SES measures and per capita solid waste generation rate, urban unemployment and urban employment were probably not good predictors of waste generation. However, some studies have observed association between per capita waste generation rate and income levels (employment provides opportunities for earning incomes) [12,53–55].

Additionally, whereas there was moderate evidence of association between urban employment and the proportion of households connected to the central sewer system, a substantially stronger evidence of association was observed between urban unemployment and proportion of households engaged in non-standard practices of liquid waste disposal. This probably meant that the implementation of Ghana’s poverty reduction strategies (GPRS) without consideration to bridge urban unemployment gaps could exacerbate the widening urban health inequalities [2,17,18,40].

## 5. Conclusions

While some of the area-based socioeconomic measures alone were not valid proxies of SES, others were valid at aggregate levels. And on the whole, aggregating the area-based socioeconomic measures into a uni-dimensional attribute and generating wealth quintiles from the uni-dimensional attribute was observed to more robustly predict SES and therefore a more valid measure at community level. Strong evidence of differences in neighborhood urban environmental quality existed across the wealth quintiles. This observation suggested that socioeconomic conditions were important drivers of change in neighborhood urban environmental quality conditions. This probably provides clues that urban environmental interventions aimed at infectious disease prevention would benefit considerably from simultaneous implementation with social interventions if they were to be effective. We conclude that widening socioeconomic inequalities (e.g., urban unemployment, urban employment, *etc.*,) at household level could worsen the existing urban environmental health inequalities at community level. It would make sense therefore if urban environmental interventions aimed at infectious disease prevention and control, were implemented simultaneously with complementary social interventions in order to be effective.

## 6. Limitations of the study

In general, the proportions of economically active and economically inactive populations were not shown to be valid measures of the area-based socioeconomic conditions. For instance, the positive (*i.e.,* a unit increase in population economic inactivity resulted in an increase in per capita solid waste generation rate) association between economically inactive population and per capita solid waste generation (regression coefficient = 0.276) was obtuse. High values of the proportion of economically inactive population represented low socioeconomic status and high values of the proportion economically active cluster populations represented high socioeconomic status. However, with the understanding that the per capita waste generation rates in high socioeconomic areas have been theoretically reported to be higher than those from low socioeconomic areas [12,55], the observed association between neighborhood urban environmental quality conditions and the proportion of economically inactive and/or active populations somewhat did not make sense. On account of this, both the proportion of economically active and/or inactive cluster population were regarded as probably unreliable measures/proxies of area-based SES. For instance, the fact that a resident was economically active did not mean that the individual was employable and could contribute to the community’s pool of wealth. In a similar argument, the fact that an individual was economically inactive did not mean that such individual could not generate income and/or contribute to the community’s wealth. Therefore, economically active or inactive factor did not predict community income or wealth and probably invalid proxy measure of SES. Data attributes that might affect their validity and reliability include; data completeness and coverage, misclassification and reporting biases. The Ghana Census covers the entire population and approximately 100 percent complete. In addition, Ghana’s population is fairly well defined and the variables enumerated were also fairly discretely defined without overlaps. Therefore both data completeness and misclassification did not present any perceived data limitation and therefore presented no perceived validity threats to the Ghana census data. However, it was possible that respondents to census questionnaire did not provide correct answers to census questions or might not have responded accurately to questions on the variables collected during the census. This meant that the Ghana census data might be prone to reporting bias which might have affected the results and conclusions of this study.

### 

#### What is already known about this subject:

The influence of socioeconomic status (SES) on health inequalities is already widely known globally.

#### What this study adds:

- Adds to the limited literature on the influence of area-based urban socioeconomic conditions on neighborhood environmental quality in a rapidly urbanizing low income community in Africa
- Establishes the evidence of the relationship between area-based socioeconomic conditions and urban neighborhood environmental quality.
- Showed strong evidence of differences in neighborhood urban environmental quality across urban wealth gradients but that some components of urban environmental quality had no association with the contextual socioeconomic conditions
- Suggests that widening socioeconomic inequalities (e.g., urban unemployment, income gaps, *etc.*) at household level could worsen the existing urban environmental health inequalities at community level.

## Figures and Tables

**Table 1.** Socioeconomic classes and environmental health inequality.

***Environmental Variable***	***SES Quintile***	***Mean***	***Coef.***	***Std. Err.***	***p-value***	***95%CI***	
Total waste generated (kg)	Poorest	2,970	5,170	2,742	0.064	−307	10,647
	Lower Middle Class	8,140	9,156	2,787	0.002	3,588	14,723
	Middle Class	12,126	13,748	2,787	0.000	8,180	19,315
	Upper Middle Class	16,718	8,439	2,838	0.004	2,769	14,108
	Richest	11,409	−	−	−	- -	
Per cap waste generation (kg/person/day)	Poorest	0.340	0.067	0.040	0.103	−0.014	0.147
	Lower Middle Class	0.407	0.139	0.041	0.001	0.057	0.220
	Middle Class	0.478	0.104	0.041	0.014	0.022	0.186
	Upper Middle Class	0.444	0.110	0.042	0.010	0.027	0.194
	Richest	0.450	-	-	-	- -	
Proportion of waste collected (%)	Poorest	0.073	−0.111	0.044	0.403	−0.057	0.139
	Lower Middle Class	0.069	−0.217	0.045	0.044	0.003	0.201
	Middle Class	0.089	−0.238	0.045	0.016	0.023	0.222
	Upper Middle Class	0.195	−0.233	0.046	0.023	0.017	0.219
	Richest	0.306	-	-	-	- -	
Proportion of waste uncollected (waste deposition) (%)	Poorest	0.427	0.041	0.049	0.015	−0.110	−0.023
	Lower Middle Class	0.432	0.102	0.041	0.000	−0.308	−0.127
	Middle Class	0.411	0.123	0.050	0.000	−0.328	−0.148
	Upper Middle Class	0.350	0.118	0.051	0.000	−0.325	−0.142
	Richest	0.309	-	-	-	- -	
Proportion households using sewer disposal (%)	Poorest	0.047	−0.193	0.039	0.000	−0.271	−0.115
	Lower Middle Class	0.041	−0.227	0.040	0.000	−0.307	−0.148
	Middle Class	0.067	−0.253	0.040	0.000	−0.333	−0.174
	Upper Middle Class	0.101	−0.246	0.041	0.000	−0.327	−0.166
	Richest	0.294	-	-	-	- -	
Proportion of households using non-sewer disposal (%)	Poorest	0.453	0.099	0.036	0.008	0.027	0.171
	Lower Middle Class	0.459	0.112	0.037	0.003	0.038	0.185
	Middle Class	0.433	0.137	0.037	0.000	0.064	0.211
	Upper Middle Class	0.421	0.131	0.038	0.001	0.056	0.206
	Richest	0.322	-	-	-	- -	
Proportion of households using pit latrine services (%)	Poorest	0.032	−0.008	0.011	0.454	−0.029	0.013
	Lower Middle Class	0.024	−0.012	0.011	0.273	−0.033	0.010
	Middle Class	0.020	0.013	0.011	0.231	−0.008	0.034
	Upper Middle Class	0.045	−0.001	0.011	0.950	−0.022	0.021
	Richest	0.031	-	-	-	- -	
Proportion of household using bucket/pan latrine services (%)	Poorest	0.043	0.010	0.018	0.573	−0.025	0.045
	Lower Middle Class	0.053	0.020	0.018	0.278	−0.016	0.055
	Middle Class	0.063	0.028	0.018	0.127	−0.008	0.063
	Upper Middle Class	0.071	0.001	0.018	0.949	−0.035	0.038
	Richest	0.044	-	-	-	- -	
Proportion of households using facility in different house (%)	Poorest	0.071	−0.021	0.009	0.021	−0.039	−0.003
	Lower Middle Class	0.050	−0.028	0.009	0.003	−0.046	−0.010
	Middle Class	0.043	−0.025	0.009	0.007	−0.043	−0.007
	Upper Middle Class	0.046	−0.026	0.009	0.005	−0.045	−0.008
	Richest	0.044	-	-	-	- -	
Proportion of households using public toilet services (%)	Poorest	0.206	0.101	0.040	0.013	0.022	0.180
	Lower Middle Class	0.149	0.133	0.040	0.002	0.052	0.213
	Middle Class	0.186	0.096	0.116	0.020	0.015	0.176
	Upper Middle Class	0.155	0.152	0.134	0.000	0.071	0.234

	Richest	0.054	-	-	-	- -	

## Appendix 1. Exploration of SES using Principal component analysis.

**Table:**

SES Variable	Mean	Std dev	Factor score
**Residents’ economic activity status**			
Economically inactive	0.610	0.076	0.500
Employed	0.139	0.041	0.500
**Residents’ educational attainment**			
No education	0.163	0.062	0.095
Pre-school education	0.044	0.008	0.448
Primary education	0.165	0.027	0.520
Middle/JSS education	0.165	0.027	0.520
Secondary/SSS education	0.155	0.032	0.132
Vocational/technical/commercial education	0.076	0.018	0.160
Post secondary education	0.029	0.009	−0.053
Residents with tertiary education	0.076	0.096	−0.451
**Residents’ occupation**			
Administrative and managerial occupations	0.147	0.064	0.419
Clerical and related occupations	0.014	0.016	0.459
Sales occupations	0.135	0.029	0.104
Service occupations	0.233	0.075	−0.490
Agriculture/husbandry/forestry/fishing/hunting occupation	0.122	0.067	0.356
Production/transport and equipment operators and laborers	0.042	0.047	−0.143
Proportion of other laborers not elsewhere classified	0.070	0.019	−0.415
Professional technical and related workers	0.237	0.047	−0.207
**Residents’ place of work**			
Residents working in agriculture hunting and forestry	0.042	0.014	−0.027
Residents working in fishing	0.029	0.041	−0.065
Residents working in mining and quarrying	0.018	0.009	0.020
Residents working in manufacturing	0.169	0.031	−0.415
Residents working in electricity gas and water supply	0.008	0.004	0.036
Residents working in construction	0.083	0.041	0.015
Residents working in wholesale/retail trade/vehicle repairers	0.264	0.081	−0.483
Residents working in hotels and restaurants	0.024	0.009	−0.071
Residents working in transport storage and communications	0.093	0.026	−0.320
Residents working in banking & finance	0.019	0.009	0.164
Residents working in real estate renting and business activities	0.041	0.016	0.217
Residents working in public administration/defense/social security	0.074	0.087	0.357
Residents working education sector	0.036	0.036	0.231
Residents working in health and social services	0.019	0.032	0.245
Residents working in other community social and personal services	0.048	0.009	−0.059
Residents working in private households	0.026	0.027	0.401
Proportion of new workers seeking employment	0.007	0.008	0.024
**Residents’ marital status**			
Married residents	0.394	0.054	0.446
Residents living together but not married	0.043	0.025	0.446
Residents separated	0.018	0.008	0.269
Residents divorced	0.027	0.017	0.427
Residents widowed	0.016	0.008	0.410
Singles	0.502	0.076	−0.424
**Residents’ ethnicity**			
Akan group	0.439	0.106	0.109
Ga Dangme group	0.267	0.164	−0.417
Ewe group	0.153	0.076	0.249
Guan group	0.031	0.013	0.396
Gurma group	0.011	0.025	0.182
Mole-Dagbani group	0.056	0.034	0.408
Grusi group	0.024	0.012	0.413
Mande group	0.008	0.009	0.369
All other ethic groups	0.013	0.021	0.298

## Appendix 2. Results of multi-variable SES included in the final PCA model.

**Table:**

SES Variable	Mean	Std dev	Factor score
Economically active	0.611	0.077	0.201
Employed	0.861	0.041	−0.131
Pre-school education	0.044	0.008	0.219
Primary education	0.165	0.027	0.305
Middle/JSS education	0.165	0.027	0.305
Residents with tertiary education	0.076	0.096	−0.319
Administrative and managerial occupations	0.014	0.016	−0.317
Clerical and related occupations	0.135	0.029	0.068
Service occupations	0.122	0.067	−0.256
Agriculture/husbandry/forestry/fishing/hunting occupation	0.042	0.047	0.113
Proportion of other laborers not elsewhere classified	0.237	0.047	0.184
Residents working in manufacturing	0.169	0.031	0.285
Residents working in wholesale/retail trade/vehicle repairers	0.264	0.081	0.296
Residents working in transport storage and communications	0.093	0.026	0.266
Residents working in public administration/defense/social security	0.074	0.087	−0.228
Residents working in private households	0.026	0.027	−0.312

## Appendix 3. PCA output showing components produced.

**Table:**

Component	Eigenvalue	Difference	Proportion	Cumulative
Comp1	5.42693	2.63348	0.3392	0.3392
Comp2	2.79345	0.71620	0.1746	0.5138
Comp3	2.07725	0.35214	0.1298	0.6436
Comp4	1.72511	0.55671	0.1078	0.7514
Comp5	1.16841	0.40396	0.0730	0.8244
Comp6	0.76444	0.10618	0.0478	0.8722
Comp7	0.65827	0.10866	0.0411	0.9134
Comp8	0.54960	0.26207	0.0344	0.9477
Comp9	0.28753	0.08384	0.0180	0.9657
Comp10	0.20369	0.08221	0.0127	0.9784
Comp11	0.12149	0.02328	0.0076	0.9860
Comp12	0.09820	0.03019	0.0061	0.9921
Comp13	0.06801	0.03279	0.0043	0.9964
Comp14	0.03522	0.01284	0.0022	0.9986
Comp15	0.02238	0.02238	0.0014	1.0000
Comp16	1.110e−16	0.00000	0.0000	1.0000
